# Serological evidence of human exposure to foodborne zoonotic parasites in Romanian patients and possible association with food habits and hygiene

**DOI:** 10.1016/j.fawpar.2024.e00240

**Published:** 2024-08-03

**Authors:** Violeta Briciu, Zsuzsa Kalmár, Anca Ieremia, Mihaela Lupșe, Mirela Flonta, Monica Muntean, Cristina Cismaru, Melinda Horvat, Amanda Rădulescu, Adriana Topan, Cristian Jianu, Angela Monica Ionică

**Affiliations:** a Clinical Hospital of Infectious Diseases of Cluj-Napoca, 400348 Cluj-Napoca, Romania; b Department of Infectious Diseases and Epidemiology, “Iuliu Hațieganu” University of Medicine and Pharmacy, 4000348 Cluj-Napoca, Romania; c Department of Microbiology, Immunology, and Epidemiology, University of Agricultural Sciences and Veterinary Medicine of Cluj-Napoca, 400372 Cluj-Napoca, Romania

**Keywords:** Foodborne parasites, Food habits, Hygiene, Romania, Seroprevalence

## Abstract

Humans may become accidental dead-end hosts for a series of zoonotic foodborne parasites, of which *Toxoplasma gondii*, *Echinococcus* spp., *Toxocara* spp., and *Trichinella spiralis* are of major public health interest, due to their potential pathological implications. The aims of the study were to evaluate the exposure to these pathogens in north-western Romania, and to investigate their potential association to risk factors. From June 2022 to January 2024, 554 patients admitted to a tertiary hospital in north-western Romania were screened for the presence of IgG antibodies against *T. gondii*, *Echinococcus* spp., *Toxocara canis*, and *T. spiralis* by ELISA, and potential risks were assessed using a questionnaire. Overall, 225 samples (40.6%) were positive for at least one pathogen. The highest seroprevalence for IgG was found for *T. gondii* (33.9%), followed by *Echinococcus* spp. (9.1%), *T. spiralis* (2.9%)*,* and *T. canis* (1.1%). For *T. spiralis*, raw meat consumption was associated with positivity. For *T. gondii,* increased age, rural environment, contact with cats, consumption of unwashed fruits/vegetables and drinking water from unverified sources were significantly associated to seropositivity. The present study provides new insights into the epidemiological status of zoonotic foodborne parasite in Romania, underlining the need to increase awareness on the importance of water, sanitation and food habits in relation with this neglected pathology.

## 1 Introduction

Despite the significant advances in medical sciences, parasitic infections still may cause considerable health problems and economic losses. The persistence and dissemination of such infections may have several causes, among which contamination of food and drinking water, poor level of hygiene, inappropriate management of human and animal excreta, as well as intensity of international travelling (Wójcik and Błaszkowska, 2013). Humans may become paratenic or accidental dead-end hosts for a series of zoonotic food-borne parasites, of which *Toxoplasma gondii*, *Echinococcus* spp., *Toxocara* spp., and *Trichinella spiralis* are of major public health interest. Infection occurs by ingestion of infective parasitic elements, either directly, or through consumption of contaminated water or food items (Despommier, 1983; Cook et al., 2000; Taira et al., 2004; Nijsse et al., 2015; Romig et al., 2017).

According to a report of the Food and Agriculture Organization of the United Nations/World Health Organization on risk management of food-borne parasites, *T. gondii*, *Echinococcus* spp., and *T. spiralis* are found in the first 7 positions using a multicriteria ranking tool for scoring parasites, while *T. canis* is placed on the 20th position (Food and Agriculture Organization of the United Nations/World Health Organization (FAO/WHO), 2014). However, the European ranking of food-borne parasites based on the FAO/WHO criteria and weights, the first four positions are represented by *E. multilocularis*, *T. spiralis*, *T. gondii* and *E. granulosus* (Bouwknegt et al., 2018).

Infections by the apicomplexan protozoan parasite *T. gondii* are widely prevalent in humans and other animals worldwide. Usually, in immunocompetent individuals, toxoplasmosis remains asymptomatic and undiagnosed, or presents as an acute mononucleosis-like syndrome. However, if the primary infection occurs during pregnancy, the parasite may cause congenital infection with devastating consequences for the foetus (Fallahi et al., 2018), therefore the anti-*T. gondii* antibodies are investigated in pregnant women, and acute infection is treated. Otherwise little data is available with regards to the seroprevalence in the general population. A recent systematic review indicated an overall seroprevalence of anti-*T. gondii* IgG of 32.1% in Europe between 2000 and 2020, with most cases being of food-borne, and not congenital origin (Calero-Bernal et al., 2023). The epidemiological history of toxoplasmosis in Romania has been thoroughly reviewed by Dubey et al. (2014). Since then, a single study focused on the general population has been published (Olariu et al., 2015).

Cystic echinococcosis (CE) is a neglected parasitic zoonosis caused by infection with the cestode *Echinococcus granulosus* sensu lato species complex. Human CE is a chronic, disabling condition, its clinical manifestations ranging from asymptomatic infection to extremely severe disease, when the size, and localization of the hydatid cyst(s) have impaired the functionality of target organs (Romig et al., 2017). Epidemiological studies performed on livestock animals seem to indicate hyperendemicity in Romania, with a prevalence of 32.34% in cattle, and 49.87% in sheep (Mitrea et al., 2012). In humans, between 1991 and 2010, the mean annual incidence ranged between 4.39 and 5.7 cases/100000 inhabitants in the western half of the country (Tamarozzi et al., 2020). The level of exposure to this parasite was recently evaluated by serology in western Romania (Păduraru et al., 2023). Alveolar Echinococcosis (AE), is caused by infection with *E. multilocularis*. In humans, the primarily affected organ is the liver, and in immunocompetent individuals, the metacestodes grow slowly, with the first clinical symptoms occurring after 5–15 years post-infection, showing features similar to malignant tumours (Gottstein et al., 2015). The parasite was first detected in rodents in Romania between 1991 and 1995, while a subsequent evaluation of foxes, revealed a prevalence ranging between 2.77 and 12.7% in the north-western part of the country (Sikó et al., 2011).

*Toxocara canis* is a zoonotic nematode, affecting predominantly young dogs (Barutzki and Schaper, 2013). Human toxocariasis is considered as the most frequent helminthozoonosis in the developed countries (Overgaauw and van Knapen, 2013). *T. canis* larvae can live up to 10 years in the human body after infection, while maintaining the ability to continue migration. Clinically manifest human toxocariasis can present in four major forms: ‘visceral larva migrans’, ‘ocular larva migrans’, neurotoxocariasis (neural larva migrans) and covert toxocariasis (Fan et al., 2015). According to the Center for Disease Control and Prevention (CDC), most infected people are asymptomatic, children and teenagers being more likely to be positive (Center for Disease Control and Prevention (CDC), 2024). Currently, data on this disease in humans from Romania is scarce, but the prevalence in dogs seems to be relatively high (26.9%) (Mircean et al., 2012).

*Trichinella* spp. are among the most globally widespread foodborne zoonotic pathogens, with infections having been diagnosed in numerous species of vertebrates (Pozio and Murrell, 2006). The severity of the disease largely depends on the number of ingested larvae, and can range from asymptomatic to fatal outcome if left untreated (Gottstein et al., 2009). The typical clinical picture of trichinellosis is characterized by fever, gastroenteritis, malaise, myalgia, facial edema, headaches, or conjunctival haemorrhages. However, diagnosis can be difficult in low-level infections, due to clinical manifestations that overlap with other diseases (Dupouy-Camet and Bruschi, 2007). Trichinellosis in humans is strictly related to food practices, as it can be acquired exclusively by the consumption of raw or undercooked meat (Pozio, 2007). Although the number of cases seems to be decreasing in Romania, the disease is still present (European Centre for Disease Prevention and Control (ECDC), 2023), and the possibility of underreporting needs to be taken into consideration.

The aims of the present study were to provide updated epidemiological data regarding human exposure to food and water-borne zoonotic pathogens in north-western Romania, by assessing the presence of specific IgG antibodies, and to investigate their potential association to risk factors.

## 2 Materials and methods

### 2.1 Study design

The study was conducted from June 2022 to January 2024 in The Clinical Hospital of Infectious Diseases from Cluj-Napoca, situated in the north-western part of Romania. Non-COVID hospitalized patients admitted during this period were given the opportunity to participate. Each patient involved in the study signed a specific consent form and filled out a questionnaire regarding the current lifestyle, that evaluated a set of potential risk factors: residence, urban/rural/ mixed (rural and urban) environment, international travel in the past year, outdoor activities, diet, hygiene, and exposure to animals (Supplementary material). In case of minor patients, the consent forms and questionnaires were filled in and signed by the parent or legal guardian. Patients were categorized according to age, as follows: child (1 ≤ age ≤ 13 years), youth (14 ≤ age ≤ 24 years), young-aged (25 ≤ age ≤ 44 years), middle-aged (45 ≤ age ≤ 60 years), early old-aged (61 ≤ age ≤ 80 years) and elderly (age ≥ 81 years). A risk scale based on six food- and hygiene-related items was defined, with total score ranging from 0 to 6: consumption of water from unverified sources (i.e. springs, wells), unpasteurized milk, raw meat, dried or smoked meat products, unwashed raw fruits/vegetables, and hand washing before every meal. Each of the six items was scored with 0 (no risk) or 1 (risk present). Previous parasitic infections were also recorded.

### 2.2 Sample collection

A sample of venous blood of participants was performed by medical personnel, in a pre-labelled clot activator and gel tube. The blood samples were left for 30 min at room temperature, followed by centrifugation at 1500 ×*g* for 15 min in order to separate the serum from the blood clot. The sera were stored at −80 °C until analysis.

### 2.3 Serological analysis

Serum samples were screened for the following anti-pathogen IgG antibodies: *Echinococcus* spp., *T. canis*, *T. gondii*, and *T. spiralis*. The analysis were performed by enzyme-linked immunosorbent assay (ELISA) in accordance whit the manufacturer's instructions, using the following commercial kits: Serion ELISA classic *Echinococcus* IgG (Virion/Serion Diagnostics, Würzburg, Germany), Human toxocara canis antibody (TC IgG) ELISA Kit (MyBioSource, San Diego, USA), Toxo IgG (Dia.Pro Diagnostic Bioprobes, Sesto San Giovanni, Italy) and *Trichinella spiralis* IgG (Bioactiva Diagnostica GmbH, Bad Homburg vor der Höhe, Germany).

According to the manufacturers, the sensitivity is >98% in all kits, with a declared specificity of: 94.81% for *T. spiralis*; 97.1% for *Echinococcus* spp., >98% for T*. gondii*, and 100% for *T. canis*. In case of *Echinococcus* spp., the manufacturers indicate potential cross-reactivity with the rheumatoid factor (RF).

### 2.4 Statistical analysis

Statistical analysis was carried out using Epi Info™ 7.2 software (CDC, Atlanta, GA, USA). The prevalence and 95% Confidence Interval (CI) were calculated globally and also according to specific variables and groups. The differences in prevalence were assessed using the chi-squared test, and the score means were compared by ANOVA or the Kruskal-Wallis H test, according to variance homogeneity. To test for colinearity and combined effects of multiple predictors, a logistic regression was ran. A level of significance (*p*) of 0.05 was chosen.

## 3 Results

### 3.1 Study group

From the total of 554 human serum samples collected from hospitalized patients who consented to participate during the study period, 85.4% (473; 95% CI: 82.2–88.1) of the patients completed all items in the questionnaire, while 14.6% (81; 95% CI: 11.9–17.8) provided partial responses or none. The average age was of 33 ± 22.89 years, and the median age was 33.5 years. The distribution of patients according to demographic data and answers provided in the questionnaires is presented in Table 1.

**Table 1:** The distribution of patients according to demographic data, travel history, food and hygiene related habits, evaluated risk score and animal contact.

	number	%	95% CI
**Sex**			
M	276	49.8	45.7–54.0
F	278	50.2	46.0–54.3

**Age group**			
Child (0–13 years)	157	28.3	24.8–32.2
Youth (14–24 years)	48	8.7	6.6–11.3
Young-aged (25–44 years)	182	32.9	29.1–36.9
Middle-aged (45–60 years)	82	14.8	12.1–18
Early old-aged (61–80 years)	78	14.1	11.4–17.2
Elderly (>81 years)	7	1.3	0.6–2.6

**Environment**			
Rural	165	29.8	26.1–33.7
Urban	309	55.8	51.6–59.9
Mixed	27	4.8	3.4–7.0
Not specified	53	9.6	7.4–12.3
Apartment (flat)	254	45.8	41.7–50.0
House	246	44.4	40.3–48.6
Not connected to the centralized sewage system	95	17.1	14.2–20.5
Not specified	54	9.7	7.5–12.5

**International travelling during the past year**			
Yes	180	32.5	28.7–36.5
No	312	56.3	52.2–60.4
Not specified	62	11.2	8.8–14.1

**Outdoor activities**			
Never	58	10.5	8.2–13.3
Rarely	152	27.4	23.9–31.3
Once a week	75	13.5	10.9–16.6
Daily	216	39.0	35.0–43.1
Not specified	53	9.6	7.4–12.3

**Food an hygiene-related items**			
***Drinking water from unverified sources (spring/wells)***			
Yes	86	15.5	12.7–18.8
No	410	74.0	70.2–77.5
Not specified	58	10.5	8.2–13.3

***Consumption of unpasteurized milk***			
Yes	116	20.9	17.8–24.5
No	380	68.6	64.6–72.3
Not specified	58	10.5	8.2–13.3

***Consumption of raw or undercooked meat***			
Yes	61	11.0	8.7–13.9
No	435	78.5	74.9–81.7
Not specified	58	10.5	8.2–13.3

***Consumption of dried or smoked meat products***			
Yes	396	71.5	67.6–75.1
No	100	18.0	15.1–21.5
Not specified	58	10.5	8.2–13.3

***Consumption of unwashed fruit and vegetables***			
Yes	219	39.5	35.5–43.7
No	277	50	45.8–54.1
Not specified	58	10.5	8.2–13.3

***Hand washing before every meal***			
Yes	188	33.9	30.1–38.0
No	308	55.6	51.4–59.7
Not specified	58	10.5	8.2–13.3

***Risk score***			
0	28	5.0	3.5–7.2
1	137	24.7	21.3–28.5
2	158	28.5	24.9–32.4
3	100	18.0	15.1–21.5
4	55	9.9	7.7–12.7
5	15	2.7	1.6–4.4
6	3	0.5	0.2–1.6
Not calculated	58	10.5	8.2–13.3

**Contact with animals**			
Yes	289	52.8	48.0–56.3
Specifically dogs	224	40.9	36.9–45.1
Specifically cats	127	23.2	19.9–26.9
No	207	37.4	33.4–41.5
Not specified	58	10.5	8.2–13.3

### 3.2 Serology results and risk factors

In total, 225 out of 544 (40.6%) samples were positive by ELISA and 47 (8.5%) had equivocal index values for IgG antibodies for at least one pathogen (Table 2). Of the ELISA-positive sera, 191 (84.9%; 95% CI: 79.5–89.3) were positive for antibodies against a single pathogen, while 33 (14.7%; 95% CI: 10.3–20.0) for two pathogens, all of which being positive for anti-*Toxoplasma* IgG, and another pathogen, as follows: *Echinococcus* spp. in 27 samples, *T. spiralis* in four samples, and *T. canis* in two samples. One serum sample (95% CI: 0.1–2.5) was positive for three pathogens: *T. gondii, T. spiralis*, and *Echinococcus* spp. The highest seroprevalence for IgG was found for *T. gondii*, followed by *Echinococcus* spp., *T. spiralis,* and *T. canis* (Table 2).

**Table 2:** Seroprevalence of IgG antibodies against *Echinococcus* spp., *T. canis*, *T, gondii*, and *T. spiralis*. n = number.

Pathogen	Seroprevalence % (n; 95% CI)	
	Positive	Equivocal
*Echinococcus* spp.	9.1 (50; 6.9–11.7)	8.5 (47; 6.5–11.1)
*Toxocara canis*	1.1 (6; 0.5–2.4)	2.0 (11; 1.1–3.5)
*Toxoplasma gondii*	33.9 (188; 30.1–38.0)	–
*Trichinella spiralis*	2.9 (16; 1.8–4.7)	2.4 (13; 1.4–4.0)
**TOTAL**	**40.6 (225; 36.6–44.8)**	**8.5 (47; 6.4–11.1)**

For statistical analysis, all samples having equivocal index values were regarded as negative. The total seroprevalence was relatively equal among genders, as was the case also for patients residing in the rural (48.5%) and mixed (48.1%) environment. According to the age groups classification, old age was associated with a higher seropevalence for *T. gondii* and *T. spiralis* (Table 3).

**Table 3:** **Seroprevalence of anti- *Echinococcus* spp., *T. canis*, *T, gondii*, and *T. spiralis* IgG, according to gender, environment, activities, age groups, exposure to animals, travel history, and household.** n = number of positive samples, % = seroprevalence, *p < 0.05.

Categories	Seroprevalence % (n; 95% CI)				
	Pathogen				Total
	*Echinococcus* spp.	*T. canis*	*T. gondii*	*T. spiralis*	
**Gender**					
Male	8.7 (24; 5.7–12.7)	1.1 (3; 0.2–3.1)	35.1 (97; 29.5–41.1)	3.3 (9; 1.5–6.1)	**40.6 (112; 34.7–46.6)**
Female	9.4 (26; 6.3–13.5)	1.1 (3; 0.2–3.1)	32.7 (91; 27.3–38.6)	2.5 (7; 1.3–5.2)	**40.7 (113; 34.8–46.7)**

**Environment**					
Urban	8.4 (26; 5.8–12.0)	1.3 (4; 0.5–3.3)	25.9 (80; 21.3–31.1^⁎^	3.6 (11; 2.0–6.3)	**33.0 (102; 28.0–38.4)**^**⁎**^
Rural	9.8 (16; 5.7–15.5)	1.2 (2; 0.2–4.4)	41.8 (69; 34.2–49.7)	2.5 (4; 0.7–6.2)	**48.5 (80; 40.6–56.4)**
Mixed	7.4 (2; 0.9–24.3)	–	44.4 (12; 25.5–64.7)	–	**48.1 (13; 28.7–68.1)**
Not specified	11.3 (6; 4.2–23.0)	–	50.9 (27; 36.8–64.9)	1.9 (1; 0.1–10.1)	**56.6 (30; 42.3–70.2)**

**Outdoor activities**					
Never	10.3 (6; 3.9–21.2)	–	37.9 (22; 25.5–51.6)	5.2 (3; 1.1–14.4)	**46.6 (27; 33.3–60.1)**^**⁎**^
Rarely	5.3 (8; 2.3–10.1)	1.3 (2; 0.2–4.7)	30.9 (47; 23.7–38.9)	4.0 (6; 1.5–3.4)	**36.2 (55; 28.6–44.4)**
Once a week	6.7 (5; 2.2–14.9)	1.3 (1; 0.1–7.2)	22.7 (17; 13.8–33.8)	1.3 (1; 0.1–7.2)	**26.7 (20; 17.1–38.1)**
Daily	11.7 (25; 7.7–16.8)	1.4 (3; 0.3–4.0)	34.7 (75; 28.4–41.5)	2.3 (5; 0.8–5.4)	**43.1 (93; 36.4–50.0)**
Not specified	11.3 (6; 4.2–23.0)	–	50.9 (27;36.8–64.9)	1.8 (1; 0.1–10.1)	**56.6 (30; 42.3–70.2)**

**Age groups**					
Child	5.2 (8; 2.3–9.9)	1.9 (3; 0.4–5.6)	8.3 (13; 4.5–13.7)^⁎^	2.6 (4; 0.7–6.5)^⁎^	**17.2 (27; 11.7–24.0)**^**⁎**^
Youth	6.3 (3; 1.3–17.2)	–	8.3 (4; 2.3–20.0)	6.3 (3; 1.3–17.2)	**12.5 (6; 4.7–25.5)**
Young aged	11.5 (21; 7.3–17.1)	1.1 (2; 0.1–3.9)	36.8 (67; 29.8–44.3)	3.9 (7;1.6–7.8)	**44.5 (81; 37.2–52.0)**
Middle-aged	13.4 (11; 6.9–22.7)	1.2 (1; 0.1–6.6)	53.7 (44; 42.3–64.8)	1.2 (1; 0.1–6.6)	**59.8 (49; 48.3–70.4)**
Old adults	9.0 (7; 3.7–17.6)	–	70.5 (55; 59.1–80.3)	–	**73.1 (57; 61.8–82.5)**
Elderly	–	–	71.4 (5; 29.0–96.3)	14.3 (1; 0.4–57.9)	**71.4 (5; 29.0–96.3)**

**Exposure to animals**					
Exposed	9.4 (27; 6.3–13.4)	0.7 (2; 0.1–2.5)	34.6 (100; 29.1–40.4)	2.8 (8; 1.2–5.4)	**40.8 (118; 35.1–46.7)**
Exposed to dogs	9.0 (24; 5.6–13.6)	0.4 (1; 0.01–2.5)	37.5 (84; 31.1–44.2)	2.7 (6; 1.0–5.8)	**42.9 (96; 36.3–49.6)**
Exposed to cats	9.5 (12; 5.0–15.9)	1.57 (2; 0.2–5.6)	44.9 (57; 36.0–54.0)^⁎^	2.4 (3; 0.5–6.8)	**53.5 (68; 44.5–62.4)**^⁎^
Unexposed	8.2 (17; 4.9–12.8)	1.9 (4; 0.5–4.9)	29.5 (61; 23.4–36.2)	2.9 (6; 1.1–6.2)	**36.7 (76; 30.1–43.7)**
Not specified	10.3 (6; 3.9–21.2)	–	50.9 (27; 36.8–64.9)	3.4 (2; 1.1–6.9)	**53.4 (31; 41.9–68.3)**

**Travel history during the past year**					
With	7.8 (14; 4.3–12.7)	0.6 (1; 0.1–3.1)	28.3 (51; 21.9–35.5)^⁎^	3.3 (6; 1.2–7.1)	**33.3 (60; 26.5–40.7)**^**⁎**^
Without	9.7 (30; 6.9–13.5)	1.6 (5; 0.7–3.7)	34.5 (107; 29.3–39.7)	2.9 (9; 1.5–5.4)	**42.3 (132; 37.0–47.9)**
Not specified	9.7 (6; 3.6–19.9)	–	48.4 (30; 35.5–61.4)	1.6 (1; 0.1–8.7)	**52.23 (33; 40.1–66.0)**

**Household**					
House	10.3 (25; 6.7–14.8)	1.2 (3; 0.3–3.6)	40.2 (99; 34.1–46.7)^⁎^	3.2 (8; 1.4–6.1)	**46.8 (115; 40.4–53.2)**
Flat	7.5 (19; 4.6–11.4)	1.2 (3; 0.2–3.4)	24.4 (62; 19.3–30.2)	2.9 (7; 1.2–5.8)	**31.5 (80; 25.8–37.6)**
Not specified	11.1 (6; 4.2022.6)	–	50 (27; 36.1–63.9)	1.9 (1; 0.1–9.9)	**55.6 (30; 41.4–69.1)**
**Total**	**9.1 (50; 6.9–11.7)**	**1.1 (6; 0.5–2.4)**	**33.9 (188; 30.1–38.0)**	**2.9 (16; 1.8–4.7)**	**40.6 (225; 36.6–44.8)**

For *T. spiralis* (*p* = 0.04) and *Echinococcus* spp. (*p* = 0.03), raw meat consumption was significantly associated with positive serology. Statistically significant differences were also found within age groups for *T. spiralis* (*p* = 0.03), with the elderly having a higher positivity rate than the other age-group categories (Table 3).

For *T. gondii,* early old-aged adults (70.5%) and elderly people (71.4%) had a significantly higher seroprevalence compared to other age-group categories (*p* = 0.0001). Also, statistically significant higher prevalences were recorded in patients from rural and mixed environment compared to urban (*p* = 0.0001), those living in houses (*p* = 0.0001), and having contact with cats (*p* = 0.0006). Regarding the food- and hygiene related items, patients who declared that they consumed unwashed fruits/vegetables (*p* = 0.0005) and those who use unverified water sources (spring or well water, *p* = 0.001), seem to be more exposed to *T. gondii* infection (Table 4). Interestingly, a significantly lower seroprevalence (*p* = 0.0157) was found in the category of patients with international travel history in the past 12 months (Table 3). Following the logistic regression, four factors were retained as significant predictors: age, environment, consumption of raw meat, and unwashed fruits and vegetables (Table 5).

**Table 4:** **Seroprevalence of anti- *Echinococcus* spp., *T. canis*, *T, gondii*, and *T. spiralis* IgG, according to food- and hygiene-related items.** n = number of positive samples, % = seroprevalence, *p < 0.05.

Categories	Seroprevalence % (n; 95% CI)				
	Pathogen				Total
	*Echinococcus* spp.	*T. canis*	*T. gondii*	*T. spiralis*	
**Categories**					
Water					
Tap or bottled water	9.1 (37; 6.7–12.3)	1.2 (5; 0.5–2.8)	29.3 (120; 25.1–33.9)^⁎^	2.5 (10; 1.3–4.5)	**36.6 (150; 32.1–41.4)**^**⁎**^
Other sources	8.1 (7; 3.3–16.0)	1.2 (1; 0.1–6.3)	47.7 (41; 36.8–58.7)	4.7 (4; 1.3011.5)	**51.2 (44; 40.1–62.1)**

Milk					
Pasteurized	8.2 (31; 5.8–11.4)	1.6 (6; 0.7–3.4)	32.8 (38; 24.3–42.1)	2.4 (9; 1.3–4.5)	**37.9 (144; 33.2–42.9)**
Unpasteurized	11.2 (13; 6.1–18.40)	–	32.4 (123; 27.9–37.2)	4.3 (5; 1.4–9.8)	**43.1 (50; 33.9–52.6)**

Meat					
Raw	19.7 (12; 10.6–31.8)^⁎^	1.0 (4; 0.4–2.6)	49.2 (30; 36.1–62.3)	4.9 (3; 1.0–13.7)^⁎^	**57.4 (35; 44.0–70.0)**^**⁎**^
Cooked	7.4 (32; 5.3–10.3)	2.0 (2; 0.3–7.2)	30.1 (131; 26.0–34.6)	2.5 (11; 1.4–4.5)	**36.6 (159; 32.2–41.2)**
Dried/smoked	8.3 (33; 6.0–11.5)	–	34.3 (136; 29.8–39.2)	2.8 (11; 1.6–4.9)	**40.4 (160; 35.7–45.3)**

Fruits/Vegetables					
Washed	6.5 (18; 3.9–10.1)	1.4 (4; 0.4–3.7)	26.0 (72; 20.9–31.6)^⁎^	3.3 (9; 1.5–6.1)	**32.0 (91; 27.4–38.7)**^**⁎**^
Unwashed	12.0 (26; 8.0–17.1)	0.9 (2; 01–3.3)	40.6 (89; 34.1–47.5)	2.3 (5; 0.8–5.3)	**47.0 (103; 40.3–53.9)**

Hygiene (hand washing before every meal)					
yes	7.8 (24; 5.3–11.4)	1.0 (3; 0.3–2.8)	30.5 (94; 25.6–35.9)	2.6 (8; 1.3–5.1)	**36.4 (112; 31.2–41.9)**
no	10.6 (20; 6.6–16.0)	1.6 (3; 0.3–4.6)	35.6 (67; 28.8–42.9)	3.2 (6; 1.2–6.8)	**43.6 (82; 36.4–51.0)**

**Table 5:** **The effect of different predictors on the seroprevalence of *T. gondii*.** *p < 0.05.

Predictor	Odds ratio	95% CI	*Z*-statistic	*p*
Raw meat	2.49	1.32–4.72	2.8	0.005*
Unwashed fruits/ vegetables	1.63	1.03–2.59	2.07	0.038*
Household (house/apartment)	1.54	0.89–2.64	1.55	0.12
Contact with cats	1.51	0.89–2.57	1.54	0.12
Environment	1.37	1.04–1.81	2.23	0.026*
Drinking water	1.15	0.71–1.84	0.57	0.57
Age	1.05	1.04–1.07	9.02	<0.0001*
Dried or smoked meat products	0.97	0.52–1.82	0.08	0.93

For *T. canis*, no significant differences were noted regarding any of the investigated factors.

The mean risk scores were generally higher in seropositive patients, as compared to negative ones, for each pathogen, but the difference was significant only in the case of *T. gondii* (Table 6).

**Table 6:** **Seroprevalence of anti- *Echinococcus* spp., *T. canis*, *T, gondii*, and *T. spiralis* IgG, according to calculated risk scores and means comparison.** n = number of positive samples, % = seroprevalence, *p < 0.05.

Score	Seroprevalence % (n; 95% CI)				
	*Echinococcus* spp.	*T. canis*	*T. gondii*	*T. spiralis*	Total
0	3.6 (1; 0.1–18.4)	3.6 (1; 0.1–18.4)	10.7 (3; 2.3–28.2)^⁎^	3.6 (1; 0.1–18.4)	**17.9 (5; 6.1–99.2)**^**⁎**^
1	8.9 (12; 6.7–15.0)	1.5 (2; 0.2–5.3)	25.3 (35; 18.5–33.7)	1.5 (2; 0.2–5.3)	**33.6 (46; 25.7–42.1)**
2	6.3 (10; 3.1–11.3)	1.3 (2; 0.2–4.5)	33.5 (53; 26.2–41.5)	2.5 (4; 0.7–6.4)	**37.8 (60; 30.4–46.0)**
3	10 (10; 4.9–17.6)	–	32 (32; 23.0–42.1)	5.0 (5; 1.6–11.3)	**40 (40; 30.3–50.3)**
4	12.7 (7; 5.3–24.5)	1.8 (1; 0.1–9.7)	49.1 (27; 35.4–62.9)	1.8 (1; 0.1–9.7)	**52.7 (29; 38.8–66.4)**
5	20 (3; 4.3–48.1)	–	66.7 (10; 38.4–88.2)	6.7 (1; 0.2–32.0)	**80 (12; 51.9–95.7)**
6	33.3 (1; 0.8–90.6)	–	33.3 (1; 0.8–90.6)	–	**66.7 (2; 9.4–99.2)**
Not specified	10.3 (6; 3.9–21.2)	–	50 (27; 36.1–63.9)	3.4 (2; 1.1–6.9)	**53.4 (31; 40.4–65.1)**
Mean score of positive patients	3.3	1.7	3.4	3.2	**3.3**
Mean score of negative patients	2.5	2.6	2.4	2.5	**2.8**
Comparison	*F* = 2.81; *p* = 0.09	*F* = 1.31; *p =* 0.25	*F* = 14.48; *p* < 0.0001*	*H* = 4.28; *p* = 0.23	*F* = 14.38; *p* < 0.0001*

## 4 Discussion

As far as the authors are aware, this is the first and largest study from Romania that brings data on seroprevalence of four foodborne parasites from a cohort of hospitalized patients. The four infections investigated are of major concern for public health.

*Toxoplasma gondii* seroprevalence was described in 33.94% of the study group patients. A recent publication that evaluated 152 published studies determined an overall range of seroprevalence between 0.5 and 87.7%, in 648,010 subjects, with an average global seroprevalence rate of 25.7% and average seroprevalence of 29.7% in Europe (Molan et al., 2019). A single study of the general population in Romania has been published to date, reporting a seroprevalence of 64.8% (Olariu et al., 2015). A retrospective 15 years study performed in our hospital unit on 27,169 toxoplasmosis antenatal screening patients found a seroprevalence of 29.12% (Briciu et al., 2023), lower than in the present study, possibly due to the lower median age (29 years) of the pregnant women evaluated during the previous study. A statistically significant lower seroprevalence was found in children aged 0–13 years old (8.3%) compared to older group patients, lower than the seropositivity of 16.6% found in a study in children aged 0–18 years old from western Romania (Căpraru et al., 2019). An increase in seroprevalence with an increase in age was also reported in blood donors from Romania (Lupu et al., 2022). However, an increase in age could be associated to more chances of exposure and persistent positive serology after infection, and is not per se a risk factor. More exposed to infections are patients from rural and mixed (rural/urban) environment, as they might be more exposed to oocysts in the soil around the house. Patients who declared they consume only washed fruits/vegetables and those who drink water from safe sources have a lower risk of *T. gondii* infection, confirming the role of preventive measures in reducing toxoplasmosis incidence. Interestingly, a lower seroprevalence of anti-*Toxoplasma* IgG was identified in patients with international travel history in the past 12 months. That might be linked to better access to international travel in patients living in urban areas, associated to a higher income compared to the rural area in Romanian population (Romanian National Institute of Statistics, 2023a).

*Echinococcus* spp. seroprevalence was described in 9.06% of the study group, higher than the 2.8% seroprevalence obtained from 1347 blood donors from Western Romania (Păduraru et al., 2023). The highest rates of CE tend to occur in areas where sheep are raised (Moro, 2023), and Romania has the third largest sheep flock in the European Union (Salat et al., 2017). The highest prevalence in humans (8.7%) has been documented among Mongolian and Kazak pastoralist communities (Moro, 2023), but the current study may indicate a higher exposure rate in the Romanian population. Regarding human infection, the reports in Romania originate mainly from clinical surgical cases (Bart et al., 2006; Neghina et al., 2011; Cobzaru et al., 2013; Păduraru et al., 2024). Serological studies published between 1987 and 1999, indicated seroprevalence values ranging between 0 and 26%, according to sampling strategy (Neghina et al., 2010). Between 2014 and 2015, 35 out of the investigated 7461 rural subjects from Romania were diagnosed with CE by abdominal ultrasound evaluation (Tamarozzi et al., 2018). Moreover, although showing an overall decreasing trend, the disease seems to be relatively frequent in children, and the prevalence seems to increase with age (Cobzaru et al., 2013; Păduraru et al., 2024). However, Păduraru et al. (2023), found no significant differences between *Echinococcus* seropositivity and gender, area of residence, age, contact with dogs, or rearing sheep. During the current study, raw meat consumption was the only factor associated with *Echinococcus* spp. seroprevalence. However, knowing that infection occurs solely by ingestion of parasite eggs (Romig et al., 2017), and not metacestodes, this finding is regarded as coincidental, having no epidemiological significance. The serological assay used during the present study does not discriminate between antibodies against *E. granulosus* and *E. multilocularis*. The expansion of the latter, which is a highly pathogenic species, and the increase in incidence of human AE in Europe has been documented (Oksanen et al., 2016), with its presence being confirmed both in definitive (red foxes) and intermediate hosts (rodents and humans) in Romania (Sikó et al., 2011). However, the majority of human individuals exposed to infection with *E. multilocularis* eggs exhibit resistance to disease, as shown by either seroconversion to parasite-specific antigens and maintenance of a seropositive status, and/or the presence of ‘dying’ or ‘aborted’ metacestodes (Gottstein et al., 2015).

*Trichinella spiralis* seroprevalence was described in 2.9% of the study group patients. Results were quite similar to the seroprevalence of 2% reported from 1347 blood donors, a study performed in 2018 in western Romania (Pavel et al., 2022). According to the questionnaires, none of the IgG positive patients had been diagnosed and treated for trichinellosis, confirming the underdiagnosis of this zoonosis, due to its clinical manifestations that overlap those of other diseases, such as respiratory disease, gastroenteritis or skin allergies (Nemet et al., 2013). The major risk factor is consumption of raw meat. However, there were 11 seropositive patients that recognized only cooked meat consumption; probably insufficient cooking might be an explanation. According to the last published data on trichinellosis from ECDC (2023), based on national reported data, Romania, showed a significant decrease in the five-year trend from 2017 to 2021. However, a previous 30 years retrospective study from our hospital, on 566 hospitalized trichinellosis patients suggests underreporting data as, for example in 2018, 26 patients were hospitalized with acute trichinellosis (Lupșe et al., 2023), while in the ECDC report there are only 10 infected patients reported from Romania in the same year.

*Toxocara canis* seroprevalence was described in 1.1% of the study group, discordant with the 30% seroprevalence in the healthy population mentioned in a review of data published in Romanian articles before 2011, as reviewed by Neghina et al. (2011). Seroprevalence varies between countries with reported values of 7.3% in US children, 2.5% in Germany and up to 83% in the Caribbean (Overgaauw and van Knapen, 2013). No difference in seroprevalence was described between age groups in our study group, although a higher infection risk in children has been noted, which can be explained by their behaviour (closer contact with potentially contaminated soil and sandpits).

### 4.1 Food habits and hygiene

Parasites are frequently transmitted to humans through contaminated food. Despite recent declines in attributable mortality, inadequate drinking-water, sanitation and hygiene (WASH) remains an important determinant of global disease burden of foodborne parasites, especially among young children (Prüss-Ustün et al., 2019). It is important to underline that in the present study, 15.5% of the subjects declared consuming water from unverified sources.

Humans can be infected also through the consumption of raw fresh products (fruits and vegetables) that have been directly contaminated or irrigated or washed with contaminated water (Robertson et al., 2020). 39.5% of the study group patients do not regularly wash the fruits and vegetables before consumption and 55.6% don't wash their hands every time before eating.

Although no association was found between unpasteurized milk consumption and any of the investigated pathogens, the current study, with 20.9% patients consuming unpasteurized milk, underlines the persistence of this food habit as a potential public health threat.

Regarding the risk posed by inappropriate management of human excreta, the houses of 17.1% of patients are not connected to the central sewage system. According to the data provided by National Institute of Statistics in Romanian National Institute of Statistics (2023b) only up to 59.2% of the population was connected to sewage systems in 2022.

Increasing awareness and developing educational programs on the presence and risk of neglected foodborne parasitic diseases and improvement of hygiene and food habits represent major goals for the healthcare systems worldwide. The topic is of interest also in relation to travel medicine, as the risk for foodborne parasitic infections might be associated to travel to hyperendemic areas (Akdur Öztürk and Ünver, 2017), but also to travel to European countries (Robertson et al., 2020), as in the last decades, there has been a significant increase in international human mobility.

### 4.2 Strengths and limitations

As far as the authors are aware, this is the first study that brings data from Romania on the seroprevalence of four foodborne parasitic infections in the same population group. The study group is relatively large, heterogeneous, including both sexes, from different age groups, and different environments.

The main limitation is represented by the employed ELISA kits. The actual specificity may be questionable, as it was tested against a limited number of potentially cross-reacting factors. For the nematodes *T. canis* or *T. spiralis*, cross-reactivity with antigens from other helminths, such as *Ascaris* spp., *Anisakis* spp., or *Strongyloides* spp., have been previously documented (Romasanta et al., 2003; Ma et al., 2018). In areas where polyparasitism is a common occurrence, the use of Western Blot for confirmation of ELISA results is recommended (Fillaux and Magnaval, 2013). Similarly, for *Echinococcus* spp., ELISA tests using hydatid cyst fluid antigen, although having a high sensitivity, cross-reactions with other helminth species have been documented (Eckert and Deplazes, 2004). For *T. gondii*, potential interferences evaluated by the manufacturers did not include other parasitic or viral TORCH pathogens, although they are known to occur (Abu-Madi et al., 2010; Chen et al., 2019). No supplementary confirmation tests were performed for the purpose of the present study.

The questionnaires provide information on the patients' current lifestyle, which may have differed at the moment of infection, that can not be located in time. Finally, the incomplete questionnaires and potential dishonest answers regarding risk behaviour or misunderstood questions represent other limitations in understanding and interpreting possible associations between various factors and seroprevalence.

## 5 Conclusions

The present study provides new insights into the epidemiological status of foodborne parasite in north-western Romania, bringing data on the seroprevalences of four important species: *T. gondii* (33.94%), *Echinococcus* spp. (9.06%), *T. spiralis* (2.9%) and *T. canis* (1.09%). Food habits and hygiene were associated to exposure to *T. gondii* and *T. spiralis*. Our results underline the need to increase awareness of the general population and medical staff on the presence of parasitic diseases, and on the importance of inadequate drinking water, sanitation and food habits in relation with this often neglected pathology.

## Ethics approval and consent to participate

The current study was conducted in accordance with the Declaration of Helsinki, and approved by the Ethics Committee of Clinical Hospital of Infectious Diseases of Cluj-Napoca through Decision 8899/13.05.2022. All patients signed a specific informed consent form before being sampled.

## Consent for publication

The consent form included a section stating that the results will be published, but no personal data of the participants will be disclosed.

## Funding

The work was supported by a grant agency of the Romanian Ministry of Research, Innovation, and Digitization, CNCS-UEFISCDI, project number PN-III-P1–1.1-TE-2021-0519, contract TE49/2022, within PNCDI III.

## CRediT authorship contribution statement

**Violeta Briciu:** Writing – original draft, Supervision, Methodology, Investigation, Formal analysis, Conceptualization. **Zsuzsa Kalmár:** Writing – original draft, Software, Investigation, Formal analysis, Data curation. **Anca Ieremia:** Writing – review & editing, Validation, Investigation, Formal analysis, Data curation. **Mihaela Lupșe:** Writing – review & editing, Supervision, Methodology, Formal analysis, Conceptualization. **Mirela Flonta:** Writing – review & editing, Validation, Investigation, Formal analysis. **Monica Muntean:** Writing – review & editing, Investigation. **Melinda Horvat:** Writing – review & editing, Investigation. **Amanda Rădulescu:** Writing – review & editing, Investigation. **Adriana Topan:** Writing – review & editing, Investigation. **Cristian Jianu:** Writing – review & editing, Investigation. **Angela Monica Ionică:** Project administration, Methodology, Investigation, Funding acquisition, Formal analysis, Conceptualization.

## Declaration of competing interest

The authors declare they have no competing interests.

## Data Availability

The complete dataset generated and analysed during the current study is available in the Zenodo repository, doi: https://doi.org/10.5281/zenodo.11081221.

## Appendix A Supplementary data

Supplementary material **:** Additional file 1 Questionnaire.pdf: The questionnaire used during the study (translated from Romanian). Supplementary material
