# Supplementary material for: Serological evidence of human exposure to foodborne zoonotic parasites in Romanian patients and possible association with food habits and hygiene
Source: Food Waterborne Parasitol. 2024 Aug 3;36:e00240. doi: 10.1016/j.fawpar.2024.e00240 (PMC11363570; doi:10.1016/j.fawpar.2024.e00240)
Supplement: Supplementary material — : Additional file 1 Questionnaire.pdf: The questionnaire used during the study (translated from Romanian). [file mmc1.pdf]

QUESTIONNAIRE

***Name*________________________________**

Personal Numeric Code ☐☐☐☐☐☐☐☐☐☐☐☐☐

Phone number__________________________

☐ *I am the participant*

☐ *I am the parent or guardian of the participant*

Occupation _____________________________________

International travelling during the past year?

☐ no ☐ yes where ?________________________________

Persons living in the household ____________, of which children ___________

Children in collectivity (kindergarten, school, etc.) ☐ no ☐ yes

You perform professional or recreational outdoor activities?

☐ daily ☐ weekly ☐ rarely ☐ never

You live in: ☐ the city ☐ a village ☐ in the city, but spend much time in the countryside (mixed)

☐ an apartment ☐ a house

If living in a house, the sewage system is:

☐ centralized ☐ own, uncentralized

Your main source of drinking water:

☐ tap water ☐ bottled ☐ other sources (well/spring)

Do you consume raw milk ?

☐ no ☐ yes

Do you consume raw or undercooked (rare) meat ?

☐ no ☐ yes

Do you consume dried or smoked meat products, such as sausages, or pastrami, etc. ?

☐ no ☐ yes

Do you consume unwashed fruits and vegetables?

☐ no ☐ sometimes ☐ frequently ☐ almost always

Do you consume wash your hands before a meal?

☐ no ☐ sometimes ☐ frequently ☐ almost always

Do you own the following animals?

	No	Yes	Indoor access
**Dog(s)**			
**Cat(s)**			
**Hamster(s)**			
**Fish**			
**Turtle(s) or other reptiles**			
**Cage bird(s)**			
**Rabbit(s)**			
**Cow(s)**			
**Horse(s)**			
**Sheep**			
**Goat(s)**			
**Poultry**			
**Others?**			

Have you ever been diagnosed with a parasitic disease?

☐ no ☐ yes, which ? ______________________________

Were you prescribed or did you take antiparasitic medication?

☐ no ☐ yes, which product/substance?______________________________

____ _____________

Signature Date
